# High Thermoelectric Power Factor of a Diketopyrrolopyrrole-Based Low Bandgap Polymer via Finely Tuned Doping Engineering

**DOI:** 10.1038/srep44704

**Published:** 2017-03-20

**Authors:** In Hwan Jung, Cheon Taek Hong, Un-Hak Lee, Young Hun Kang, Kwang-Suk Jang, Song Yun Cho

**Affiliations:** 1 Division of Advanced Materials, Korea Research Institute of Chemical Technology, Daejeon 34114, Republic of Korea; 2 Department of Chemical Engineering and Research Center of Chemical Technology, Hankyong National University, Anseong 17579, Republic of Korea

## Abstract

We studied the thermoelectric properties of a diketopyrrolopyrrole-based semiconductor (PDPP3T) via a precisely tuned doping process using Iron (III) chloride. In particular, the doping states of PDPP3T film were linearly controlled depending on the dopant concentration. The outstanding Seebeck coefficient of PDPP3T assisted the excellent power factors (PFs) over 200 μW m^−1^K^−2^ at the broad range of doping concentration (3–8 mM) and the maximum PF reached up to 276 μW m^−1^K^−2^, which is much higher than that of poly(3-hexylthiophene), 56 μW m^−1^K^−2^. The high-mobility of PDPP3T was beneficial to enhance the electrical conductivity and the low level of total dopant volume was important to maintain high Seebeck coefficients. In addition, the low bandgap PDPP3T polymer effiectively shifted its absorption into near infra-red area and became more colorless after doping, which is great advantage to realize transparent electronic devices. Our results give importance guidance to develop thermoelectric semiconducting polymers and we suggest that the use of low bandgap and high-mobility polymers, and the accurate control of the doping levels are key factors for obtaining the high thermoelectric PF.

For decades, semiconducting polymers have been extensively studied for their application in curved or foldable electronic devices such as the organic light emitting diodes^1^,^2^, thin-film transistors^3^,^4^,^5^,^6^,^7^, and photovoltaics^8^,^9^,^10^ owing to their flexibility, light-weight, and solution processability. In particular, the current interest in wearable device technology is promoting the development of organic thermoelectric devices that can utilize residual body heat as the power source^11^,^12^,^13^,^14^,^15^,^16^,^17^,^18^,^19^,^20^,^21^,^22^,^23^,^24^,^25^. The performance of thermoelectric materials is assessed by the dimensionless figure of merit, *ZT* = *S*^2^*σT*/*κ*, where *S, σ, T*, and *κ* are the Seebeck coefficient, electrical conductivity, absolute temperature, and the thermal conductivity, respectively. As an alternative to the figure of merit, the power factor (PF), *S*^2^*σ*, has also been widely used to evaluate the performance of thermoelectric polymers. This is because thermal conductivities of conjugated polymers are considered to be low (<1 W m^−1^K^−1^), while the Seebeck coefficients and electrical conductivities can be varied across very large ranges by doping or post-treatment^11^,^12^,^13^,^14^,^15^. The thermoelectric performance of conjugated polymers has been advancing rapidly^11^,^12^,^13^,^14^,^15^,^16^,^17^. Crispin *et al*. reported that poly(3,4-ethylenedioxythiophene):tosylate (PEDOT: Tos) films exhibited PFs up to 324 μW m^−1^K^−2^ at room temperature^16^. By exposing the PEDOT:Tos film to tetrakis(dimethylamino)ethylene vapor, the oxidation level of PEDOT was accurately controlled, resulting in an optimized thermoelectric performance. Pipe *et al*. reported that PEDOT:poly(styrenesulfonate) (PSS) films exhibited PFs up to 469 μW m^−1^K^−2^ at room temperature^17^. By immersing the PEDOT:PSS film in ethylene glycol, the total dopant volume was minimized and as a result, the thermoelectric performance could be maximized. Research efforts on thermoelectric conjugated polymers have been mainly focused on PEDOT^18^, and only a few other polymer systems showed promise in terms of thermoelectric application;^6^ Poly(3-hexylthiophene) (P3HT) and poly(2,5*-*bis(3-tetradecylthiophen-2-yl)thieno[3,2-b]thiophene exhibited PFs of 26 μW m^−1^K^−2^ by doping with a ferric salt of triflimide at room temperature^11^, and of 25 ± 8 μW m^−1^K^−2^ by treatment of tridecafluoro-1,1,2,2,-tetrahydrooctyl)-trichlorosilane at room temperature^14^, respectively. Their relatively low thermoelectric performance compared to that of PEDOT systems is expected to be from a lack of research on developing thermoelectric materials and doping processes, thus, in this study, we tried to provide the requirements for high thermoelectric semiconducting polymers and demonstrated their excellent thermoelectric performance under accurately controlled doping levels. We selected and synthesized a poly(diketopyrrolopyrrole-terthiophene), PDPP3T^7^, which is considered to be one of the best performing conjugated polymer for organic thin-film transistors. The bicyclic lactam ring of the diketopyrrolopyrrole (DPP) moiety results in a highly planar structure with excellent molecular ordering. We supposed that the high mobility of DPP polymers will enhance the electrical conductivity in the thermoelectric devices, which is a desired characteristic in thermoelectric applications. In addition, the strong electron withdrawing DPP unit is easily forming low bandgap polymers via the strong intramolecular charge transfer interaction with adjacent electron donating units^26^,^27^. The low bandgap is beneficial to obtain transparent thermoelectric devices by further doping process. More importantly, we focused on the method to enhance the electrical conductivity without drastically decreasing the Seebeck coefficient of the polymer. In the case of P3HT, it requires a large quantity of dopants to obtain high electrical conductivity, however, suffers severe losses in terms of Seebeck coefficient^28^. Thus, a small quantity of dopants and high carrier mobility are required for increasing both electrical conductivity and Seebeck coefficient.

The PDPP3T polymer showed promising proprieties satisfying high mobility, low bandgap, and low doping level, and exhibited excellent PFs up to 276 μW m^−1^K^−2^ at much lower total dopant volume than P3HT films (56 μW m^−1^K^−2^). We investigate, in detail, the optical, morphological, and thermoelectric properties of polymers on the basis of doping levels, and provide new perspectives for the design of conjugated thermoelectric polymers.

## Results and Discussion

### Physical and Optical Properties

**Figure 1:**
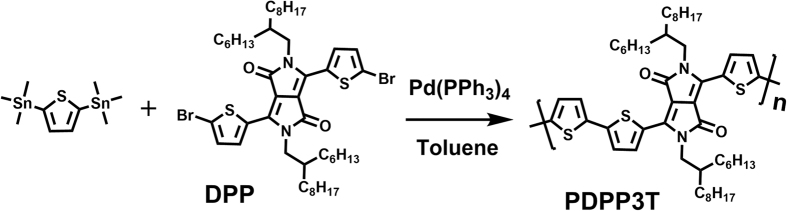
Stille polymerization for PDPP3T.

**Figure 2:**
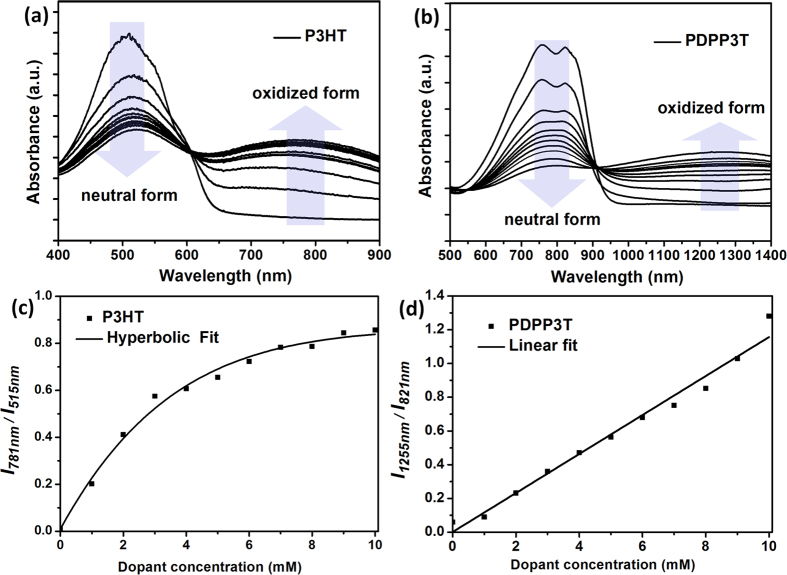
Optical absorption spectra of (**a**) P3HT and (**b**) PDPP3T films undoped and doped by spin-coating using FeCl_3_/nitromethane solutions with concentration in the range of 1–10 mM. The ratio between oxidized and neutral form of (**c**) P3HT and (**d**) PDPP3T film upon the dopant concentration.

The PF is proportional to electrical conductivity, which can be expressed as *σ* = *nqμ*, where *n, q*, and *μ* are carrier concentration, carrier charge, and carrier mobility, respectively. Therefore, the conjugated polymers with high carrier mobilities are expected to be advantageous for enhancing the thermoelectric performance. In this study, we selected P3HT and PDPP3T which are known to have high carrier mobilities in thin-film transistors. Highly regioregular P3HT with a regioregularity of 98.5% was purchased from Sigma-Aldrich, while PDPP3T polymer was synthesized via Stille polymerization of 2,5-bis(trimethylstannyl)thiophene and 3,6-bis(5-bromothiophen-2-yl)-2,5-bis(2-hexyldecyl)pyrrolo[3,4-c]pyrrole-1,4(2 H,5 H)-dione. The synthetic procedures for the polymer were summarized in Fig. 1. The synthesized PDPP3T polymer showed sizable number average molecular weights (*M*_n_) of 102,000 g/mol (polydispersity index = 2.25), determined by gel permeation chromatography using polystyrene as standard, and excellent solubility in chlorinated solvents (e.g., chloroform, chlorobenzene, and dichlorobenzene). We measured the absorption properties of P3HT and PDPP3T before (Fig. S1) and after doping (Fig. 2). The band gap of polymers was calculated from the film-state absorption onset wavelength. The measured bandgaps of P3HT (1.93 eV) and PDPP3T (1.34 eV) were quite similar with the reported bandgaps of P3HT (1.9 eV)^29^ and PDPP3T (1.3 eV)^7^. That of PDPP3T was much lower than that of P3HT. This could be attributed to the donor-acceptor structure of PDPP3T composed of the thiophene donor and DPP acceptor. This push-pull chromophore induces strong intramolecular charge-transfer interactions and large polarizability, and leads to the electron delocalization over the conjugated main chain^26^,^30^. Thus, PDPP3T can facilitate the doping process and photoconductivity. The highest occupied molecular orbital (HOMO) energy level (*E*_HOMO_) of polymers was verified by cyclic voltammetry (CV) and ultraviolet photoelectron spectroscopy (UPS). The *E*_*HOMO*_^*CV*^ was calculated from the oxidation onset potentials relative to ferrocene as an internal standard, and their cyclic voltammograms are shown in Fig. S3(c). *E*_*HOMO*_^*UPS*^ was calculated from the onset (*E*^*F*^_*VBM*_) and high binding energy cutoff (*E*_*cutoff*_) region of the polymer films, and these spectra were shown in Fig. S3(a) and (b), respectively. The *E*_*HOMO*_^*CV*^ of PDPP3T and P3HT was determined to be −5.30 and −4.97 eV, respectively, and *E*_*HOMO*_^*UPS*^ to be −4.74 and −4.31 eV, respectively. As shown in Fig. S3, the HOMO energy levels calculated from UPS showed similar trend with those of the CV measurements. Overall, PDPP3T showed much deeper HOMO and LUMO levels than P3HT.

### Thermoelectric properties

To obtain the optimal thermoelectric performance of conjugated polymers, their doping levels should be accurately controlled^11^,^12^,^13^,^14^,^15^,^16^,^17^. In this study, we demonstrate the accurate control of the doping levels of conjugated polymers via the simple and efficient post treatment of dopant solution on the polymer films. A FeCl_3_/nitromethane solution was simply over-coated by spin-coating onto the conjugated polymer films. Dopant molecules diffused into the film capture electrons from the polymer chains, thus additional rinsing and annealing process was not required for doping by spin-coating^31^. In particular, by controlling the concentration of dopant solution, the doping levels of polymer films were accurately controlled. To show the feasibility of the spin-coating method for controlling the doping level of conjugated polymers, the optical absorption spectra of the P3HT films and PDPP3T films were measured depending on the concentration of dopant solutions (Fig. 2). The relative doping levels of the P3HT films can be estimated based on the variation in their optical absorbance^31^,^32^,^33^,^34^. As shown in Fig. 2(a) and (c), the relative doping level of the P3HT film was controlled by varying the dopant concentration from 1 to 10 mM. With increasing doping level caused by the dopant uptake in the P3HT film, the absorbance for the neutral form (λ_max_ = 515 nm) decreases and the absorbance for the oxidized form (λ_max_ = 781 nm) increases. The ratio between the oxidized and neutral forms (*I*_*781nm*_/*I*_*515nm*_) showed hyperbolic response upon the dopant concentration, thus it is expected that a large amount of dopants remains undoped states in the film as the total dopant volume is increased.

**Figure 3:**
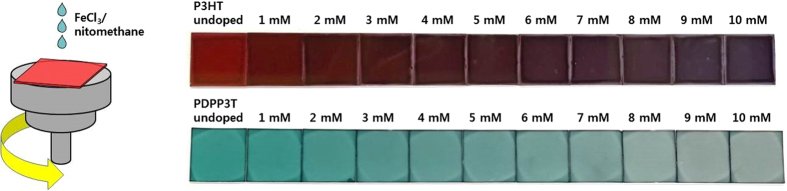
Schematic diagram of the doping by spin-coating, and photographic images of P3HT and PDPP3T films doping level controlled by varying the concentration of dopant solutions.

In case of the PDPP3T films, as the dopant concentration increases from 1 to 10 mM, the ratio between the oxidized and neutral forms (*I*_*1255nm*_/*I*_*821nm*_) linearly increased; The absorbance for the neutral form (λ_max_ = 821 nm) and the oxidized form (λ_max_ = 1255 nm) gradually decreases and increases, respectively (Fig. 2(b) and (d)). Thus, doping levels of PDPP3T films were precisely controlled by dopant concentration. In addition, the greenish color of PDPP3T film became more colorless after doping (Fig. 3) because the oxidized form of PDPP3T showed the near infra-red (IR) absorption in the range of 1000–1600 nm (Fig. S2), whereas P3HT film still exhibited strong purple absorption in 700–900 nm (Fig. 3). From the uniform color of the each doped polymer film, we can conclude that dopant molecules were uniformly doped on the whole area of the polymer film. Overall, PDPP3T was more advantageous to control the doping levels and to realize the transparent thermoelectric devices than P3HT.

**Figure 4:**
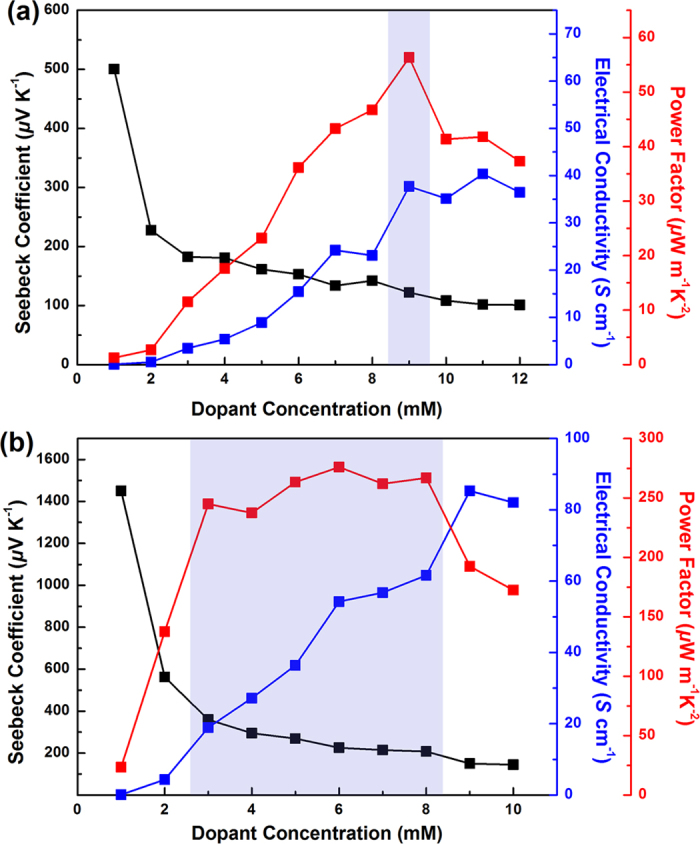
Thermoelectric properties of (**a**) P3HT and (**b**) PDPP3T doped with FeCl_3_/nitromethane solutions by spin-coating.

**Figure 5:**
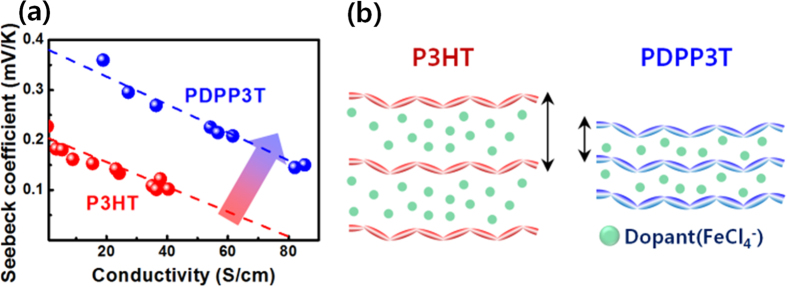
(**a**) Seebeck coefficients of the doped P3HT and PDPP3T films as a function of electrical conductivity (the dashed lines were inserted to stand out the difference of Seebeck coefficients more clearly), and (**b**) the scheme of the doped P3HT and PDPP3T with optimal thermoelectric performances.

The thermoelectric properties, i.e. the Seebeck coefficient and electrical conductivity, of the P3HT and PDPP3T films were measured as a function of the dopant concentration (Fig. 4). The Seebeck coefficient was estimated from the slope of the straight line fit of Δ*V*/Δ*T* which is measured under dark ambient conditions using a custom built system^30^. All the films exhibited positive values of Seebeck coefficient, which implies the presence of p-type characteristics in P3HT and PDPP3T. The trade-off relationship between the Seebeck coefficient and electrical conductivity of thermoelectric materials is well-known^13^,^14^,^15^,^16^,^17^,^18^,^19^. With increasing carrier concentration of thermoelectric materials, the Seebeck coefficient decreases and the electrical conductivity increases. A similar trade-off relationship was also observed in both P3HT and PDPP3T films. By the precise control of the doping levels, the optimal PFs of conjugated polymers could be obtained. At a dopant concentration of 9 mM, the P3HT film, doped by spin-coating, exhibits a peak PF value, 56 μW m^−1^K^−2^, which is higher than the previously reported PF value of P3HT film (35 μW m^−1^K^−2^) doped using the conventional immersion method^35^. Doping by spin-coating is effective not only for accurately controlling doping levels of conjugated polymers, but also for obtaining corresponding high thermoelectric PFs. Interestingly, in the case of the PDPP3T films, there is a wide range of optimal PFs. At dopant concentrations of 3–8 mM, the PFs of the PDPP3T films are in the range of 245 to 276 μW m^−1^K^−2^, which is almost 5 times higher and more uniform PFs than those of P3HT. The optimal thermoelectric properties of the P3HT and PDPP3T films doped by spin-coating are summarized in Table 1. The average PFs of the P3HT films doped with a 9 mM FeCl_3_/nitromethane solution and the PDPP3T films doped with a 6 mM FeCl_3_/nitromethane solution were 46 ± 7 and 247 ± 21 μW m^−1^K^−2^, respectively, at room temperature. The post-treatment of dopant by spin-coating was highly suitable for thin and uniform active layer of about 200 nm. We also tried to use immersion and rinsing method with the thin polymer films of P3HT and PDPP3T. The electrical conductivities of the P3HT and PDPP3T thin films doped by the immersion method and rinsed for removing the excess dopants were measured to be ~0 S/cm due to a great extent of dedoping during the rinsing process. Only thick active layer of 15 μm formed by drop-casting shows the Seebeck coefficient of 74 μV K^−1^, the electrical conductivity of 7 S cm^−1^, and the PF of 3.9 μW m^−1^K^−2^ at room temperature^36^ via the immersion and rinsing method with FeCl_3_, but this PF is significantly lower than that obtained from the post-treatment of dopant. Doping is a key factor in determining the performance of thermoelectric materials because the carrier concentration and carrier mobility of thermoelectric materials can be controlled by doping. Unlike in inorganic materials, dopants in conjugated polymers are held in position by secondary bonding, and a large number of dopants are non-ionized^17^,^28^. Pipe *et al*. reported that the carrier mobility of the conjugated polymer decreases exponentially with increasing cumulative dopant volume^17^,^28^. The decrease in the carrier mobility leads to the drop in the electrical conductivity, resulting in the poor thermoelectric PF. Thus, a smaller amount of dopants, i.e. a smaller total dopant volume, is highly important to increase the carrier mobility and Seebeck coefficient. As a result, PDPP3T, which needed smaller amount of dopants than P3HT, always showed better PFs than P3HT at the identical electrical conductivity as shown in Fig. 5a.

Figure S4 shows the X-ray diffraction (XRD) patterns of P3HT films for both undoped and doped with a 9 mM FeCl_3_/nitromethane solution, and PDPP3T films undoped and doped with a 6 mM FeCl_3_/nitromethane solution. The XRD peaks of both the P3HT films are assigned to (*00l*) reflections, which indicate the presence of lamellar type packing of P3HT chains. After doping with a 9 mM FeCl_3_/nitromethane solution, the *d*-spacing of the first order peak increases from 1.66 nm to 1.81 nm and the peak intensity decreases by approximately 1.3 times. Moreover, the distance along (*001*) direction is expanded and the crystallinity of P3HT chains decreases by dopant uptake. In case of P3HT, a large amount of dopant occupy a certain volume in conjugated polymers, resulting in the conformational change of conjugated polymer chains and increasing the tunneling distance for hopping between conjugated polymer chains. However, in the case of the PDPP3T films, after doping with a 6 mM FeCl_3_/nitromethane solution, the *d*-spacing increases slightly from 1.92 nm to 1.94 nm and the difference in the peak intensity is negligible. A small amount of dopant did not give negative effect to the intermolecular ordering. Thus, the results in Fig. 5a can be clearly related with the scheme in Fig. 5b, which shows that the smaller dopant volume with less interruption of molecular ordering can facilitate hoping and carrier transport process.

Figure S5 shows the atomic force microscope images of P3HT films undoped and doped with a 9 mM FeCl_3_/nitromethane solution, and PDPP3T films undoped and doped with a 6 mM FeCl_3_/nitromethane solution. The undoped P3HT and PDPP3T films have smooth surfaces. After doping with a 9 mM FeCl_3_/nitromethane solution, there are particulate aggregates on the top surface of P3HT. Owing to the tendency of dopants to aggregate, particulate aggregates can form on the surface of doped conjugated polymer films^13^,^14^,^15^,^37^,^38^. The relatively smaller size and number of the particulate aggregates on top of the doped PDPP3T surface can be considered as evidence of the smaller total dopant volume in the doped PDPP3T film.

Because of the limitation in precise measurement of the in-plane thermal conductivity of a thin film on a substrate, the thermal conductivities of the polymer films could not be measured. In general, the thermal conductivity, *κ*, of materials is expressed as a sum of the electronic thermal conductivity, *κ*_*e*_, and the lattice thermal conductivity, *κ*_*l*_ (*κ* = *κ*_*e*_ + *κ*_*l*_). Using the Weidemann-Franz relation (*κ*_*e*_ = *L*_*0*_*σT*, where *L*_*0*_ is Lorentz constant of 2.45 × 10^−8^ V^2^ K^−2^, *σ* is the electrical conductivity, and *T* is the absolute temperature), the electronic thermal conductivity can be calculated. The calculated electronic thermal conductivities of P3HT films doped with a 9 mM FeCl_3_/nitromethane solution and PDPP3T films doped with a 6 mM FeCl_3_/nitromethane solution are 0.031 and 0.038 W m^−1^K^−1^, respectively. The calculated electronic thermal conductivities are very low and the total in-plane thermal conductivities should be measured in the future.

## Conclusion

In this study, we report outstanding PF of 276 μW m^−1^K^−2^ in PDPP3T polymer films. The post treatment of dopant on the PDPP3T films offers simple and accurate control of the doping levels. Depending on the concentration of dopant solutions, the neutral and oxidized form of PDPP3T are precisely tuned and the low bandgap PDPP3T polymer became more colorless due to the strong absorption in near IR area. In particular, the high-mobility of PDPP3T polymer enables to enhance both electrical conductivity and Seebeck coefficient at a low level of total dopant volume. The use of low band gap and high-mobility polymers and the accurate control of the doping levels might be the key factors for obtaining the enhanced performance of thermoelectric polymers. Currently, we are studying several high-mobility and low bandgap semiconducting polymers to find structure-property relationship in thermoelectric application.

## Methods

### Synthesis of 2,5-bis(trimethylstannyl)thiophene

Thiophene (1.40 g, 16.7 mmol) was dissolved in anhydrous THF (100 mL), and then 2.5 M *n*-BuLi (14 mL, 35 mmol) was added dropwise to the solution at −78 °C. The mixture was stirred for 1 hr at room temperature and then cooled again to −78 °C. The 1 M trimethyltin chloride in hexanes (35 mL) was added dropwise and stirred overnight at room temperature. The mixture was poured into water (100 mL) and extracted with diethyl ether. The organic layer was washed three times with water and dried over magnesium sulfate. After drying the solvent, the residue was purified by recrystallization from methanol to give a white crystal (4.5 g, 66%). ^1^H NMR (400 MHz, CDCl_3_) δ 7.38 (s, 2H), 0.37 (s, 18H).

### Polymerization procedure for PDPP3T

A mixture of 2,5-bis(trimethylstannyl)thiophene (0.231 g, 0.564 mmol), 3,6-bis(5-bromothiophen-2-yl)-2,5-bis(2-hexyldecyl)pyrrolo[3,4-c]pyrrole-1,4(2 H,5 H)-dione (0.512 g, 0.564 mmol) and Pd(PPh_3_)_4_ (13 mg, 2 mol%), tetrakis(triphenylphosphine)palladium, was dissolved in 10 mL of toluene. The mixture was degassed for 30 min and then heated at 120 °C for 16 hr. After cooling to room temperature, the mixture was added to methanol. The precipitate was dissolved in chloroform and filtered with Celite to remove the metal catalyst. The polymer fibers were washed by Soxhlet extraction with methanol, acetone, hexanes and chloroform. The final polymer was obtained after reprecipitation with methanol, yielding 390 mg (83%). ^1^H NMR (400 MHz, CDCl_3_) δ 8.94 (br, 2H), 7.04 (br, 4H), 4.02 (br, 4H), 1.92 (br, 2H), 1.26 (br, 48H), 0.86 (br, 12H). Anal. Calcd for C_50_H_72_N_2_O_2_S_3_: C, 72.41; H, 8.75; N, 3.38; O, 3.86; S, 11.60, Found: C, 71.5; H, 8.7; N, 3.2, S, 10.9.

### Characterization

^1^H NMR and ^13^C NMR spectra were recorded on a Bruker Ascend^TM^-400 spectrometer, with tetramethylsilane as an internal reference. The absorption spectra were measured on a SHIMADZU/UV-2550 model UV-visible spectrophotometer. Cyclic voltammetry was performed on a BAS 100B/W electrochemical analyzer with a three-electrode cell in a 0.1N Bu_4_NBF_4_ solution in acetonitrile at a scan rate of 50 mV/s. The polymer film was coated onto a Pt wire electrode by dipping the electrode into a polymer solution in chloroform. All measurements were calibrated against an internal standard of ferrocene (Fc), the ionization potential (IP) value of which is −4.8 eV for the Fc/Fc + redox system. The number- and weight-average molecular weights of the polymers were determined by gel-permeation chromatography (GPC) with a Waters 2690 Associates liquid chromatography instrument equipped with a Waters 2414 differential refractometer. Chloroform was used as the eluent and polystyrene as the standard. The surface morphology of polymer films was measured by atomic force microscopy (AFM) using tapping mode, and the AFM scan images (2 μm × 2 μm) were acquired in tapping mode on a Nanoscope instrument (Bruker).

### Preparation of polymer thin films

P3HT (*M*_w_ 38,000 g mol^−1^, regioregularity 98.5%) was dissolved in *o*-dichlorobenzene with a concentration of 1.5 wt%. PDPP3T was dissolved in a mixture of chloroform and chlorobenzene (1:1 wt ratio) with a concentration of 1.5 wt%. The solutions were filtered with 1 μm PTFE filter. Prior to the deposition of the polymers, the substrate was cleaned consecutively by ultrasonication with a detergent solution, acetone, and isopropyl alcohol. The substrate was then dried and treated with a UV-ozone plasma for 5 min. For the surface modification, hexamethyldisilazane was spin-coated onto the glass substrate at 4000 rpm for 30 s, and annealed at 125 °C for 10 min. Conjugated polymer films were deposited onto glass substrates by spin-coating. The thickness of all polymer thin films was controlled to be 200 nm. For doping by spin-coating, FeCl_3_/nitromethane solutions were spin-coated at 3000 rpm for 30 s onto the as-prepared polymer films. Because of the very small thickness of the polymer films, approximately 200 nm, relatively short time scale is required for the dopant diffusion and doping. If the thickness of polymer films is thicker, different optimal time for the dopant diffusion and doping will be required.

### Ultraviolet photoelectron spectroscopy (UPS)

A set of samples were prepared onto ITO glass via the identical method with ‘Preparation of polymer thin films’. The polymer films were analyzed using the AXIS Ultra DLD model (KRATOS Inc.) in Korea Basic Science Institute (KBSI), Daejeon. The He I (21.2 eV) emission line was employed as a UV source. The helium pressure in the analysis chamber during analysis was about 4.0 × 10^−8^ Torr. The HOMO energy level was determined using the incident photon energy, *hv* = 21.2 eV, *E*_*cutoff*_, and *E*_*onset*_.

### Measurements

The Seebeck coefficient was measured in a vacuum chamber with a relatively low vacuum of 10^−2^ torr to minimize the effects of heat convection and conduction on the thermoelectric measurement utilizing a custom-built system^39^. The temperature difference between the both ends of the sample is controlled using Peltier devices, which are elaborately controlled by a Keithley 2200 power source and a Keithley 2460 source meter. Two pairs of T-type thermocouples with a diameter of 1 mm are employed to detect and control the temperature of the surface of sample and Peltier devices. Seebeck voltage and short circuit current are respectively measured using Keithley 2182 A nanovoltmeter and Keithley 6485 picoammeter. The gold probe tip with a diameter of 5 μm is employed to evaluate thermoelectrical characteristics of samples. Prior to the measurement, gold electrodes were deposited onto the polymer films through a shadow mask by using thermal evaporation. Two electrodes, 3 mm in width, were separated by a distance of 10 mm. The samples were placed on top of two Peltier plates. The temperature gradient between the two electrodes was varied between 1 to 10 °C by applying an electrical current through the Peltier plates. The Seebeck coefficient was estimated from the slope of the straight line fit of Δ*V*/Δ*T* (Fig. S6). To confirm the accuracy of the Seebeck coefficient measurement, the Seebeck coefficient of a commercial constantan whose Seebeck coefficient is known to be −35 μV K^−1^ was also measured (Fig. S7). The electrical conductivity was measured by the standard four-point probes for the local measurement. For measurement of the electrical conductivity, the probe tips separated by a distance of 1 mm were contacted onto the center of the films. All measurements were performed at room temperature using a Keithley 195 A digital multimeter and a Keithley 220 programmable current source.

## Additional Information

**How to cite this article**: Jung, I. H. *et al*. High Thermoelectric Power Factor of a Diketopyrrolopyrrole-Based Low Bandgap Polymer via Finely Tuned Doping Engineering. *Sci. Rep.* **7**, 44704; doi: 10.1038/srep44704 (2017).

**Publisher's note:** Springer Nature remains neutral with regard to jurisdictional claims in published maps and institutional affiliations.

## Supplementary Material

Supplementary Information

## Figures and Tables

**Table 1:** Thermoelectric properties of P3HT and PDPP3T doped by spin-coating.

Polymer/concentration of doping solution	Seebeck coefficient [μV K^−1^]		Electrical conductivity [S cm^−1^]		Power factor [μW m^−1^K^−2^]	
	Average	Maximum	Average	Maximum	Average	Maximum
P3HT/9 mM	105 ± 12	122	42 ± 3	45	46 ± 7	56
PDPP3T/6 mM	217 ± 8	226	52 ± 3	55	247 ± 21	276
