# Supplementary material for: High Thermoelectric Power Factor of a Diketopyrrolopyrrole-Based Low Bandgap Polymer via Finely Tuned Doping Engineering
Source: Sci Rep. 2017 Mar 20;7:44704. doi: 10.1038/srep44704 (PMC5357796; doi:10.1038/srep44704)
Supplement: Supplementary Information [file srep44704-s1.doc]

**Supplementary Information**

**High Thermoelectric Power Factor of a Diketopyrrolopyrrole-Based Low Bandgap Polymer via Finely Tuned Doping Engineering**

In Hwan Jung^1,+^, Cheon Taek Hong^1,+^, Un-Hak Lee^1^, Young Hun Kang^1^, Kwang-Suk Jang^2,*^, Song Yun Cho^1,*^

^1^ Division of Advanced Materials, Korea Research Institute of Chemical Technology, Daejeon 34114, Republic of Korea

^2^ Department of Chemical Engineering, Hankyong National University, Anseong 17579, Republic of Korea

**Fig. S1.** Absorption spectra of P3HT and PDPP3T in solution and film.

**Fig. S2.** Near IR absorption spectra of PDPP3T film by doping concentration.


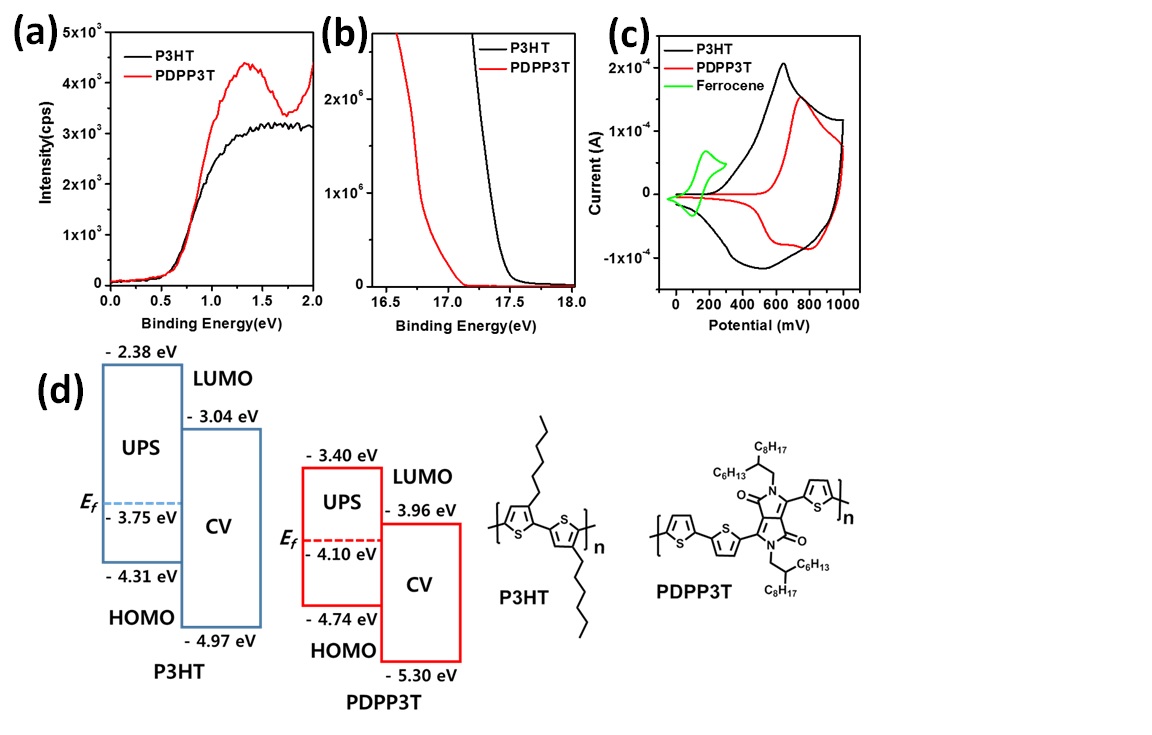


**Fig. S3**.UPS spectra of (a) the onset binding energy (*E^F^_VBM_*) and (b) high binding energy cutoff (*E_cutoff_*), (c) cyclic voltammograms, and (d) HOMO/LUMO levels of P3HT and PDPP3T.


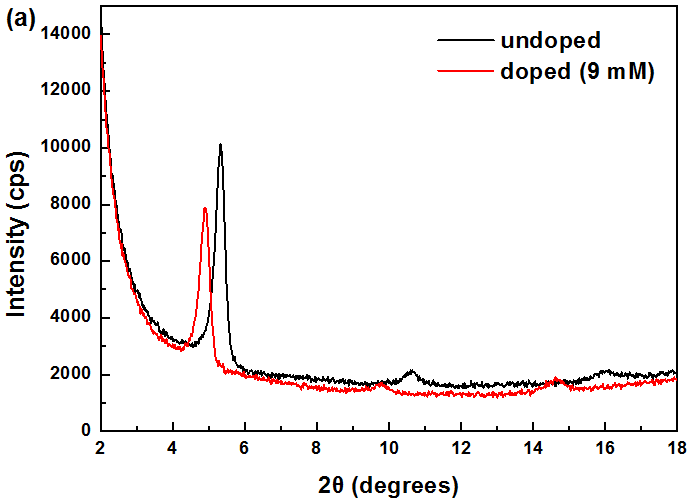

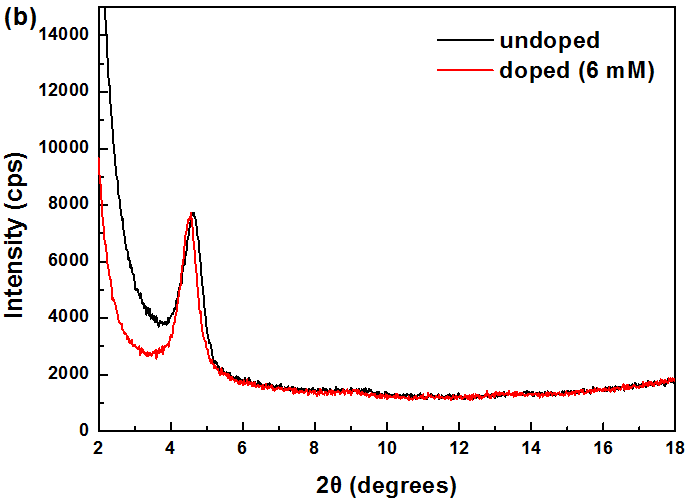


**Fig. S4.** XRD patterns of (a) P3HT films undoped and doped with a 9 mM FeCl_3_/nitromethane solution and (b) PDPP3T films undoped and doped with a 6 mM FeCl_3_/nitromethane solution.


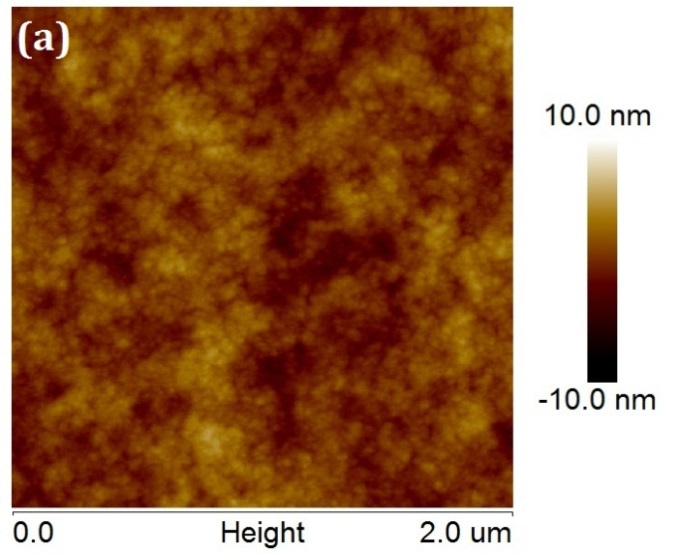

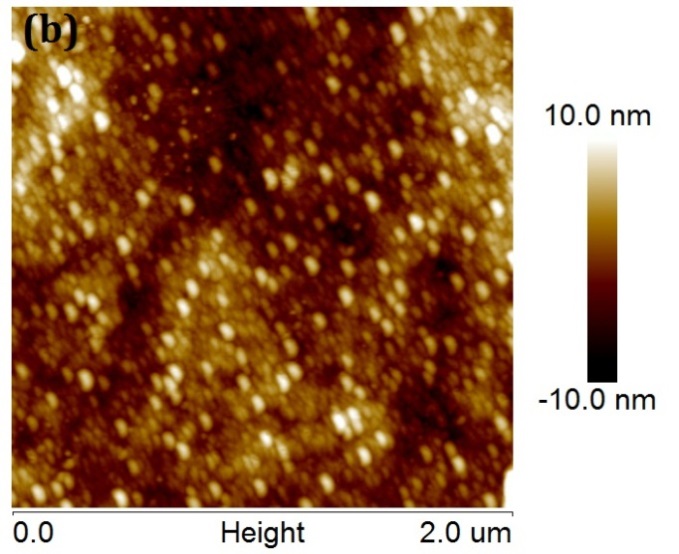

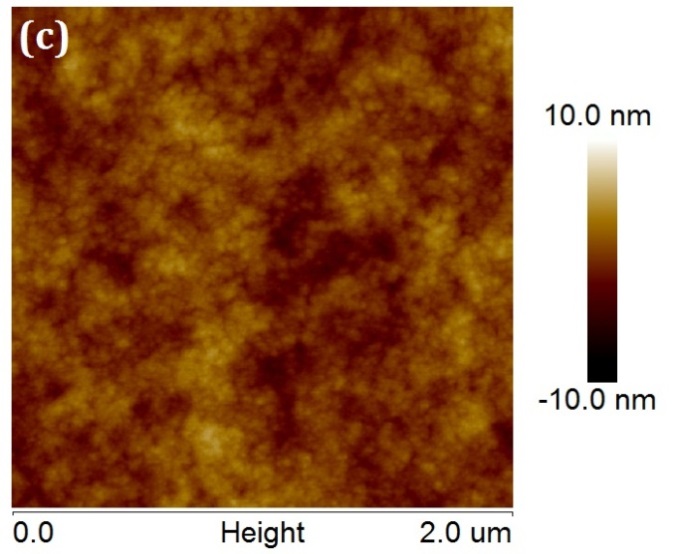

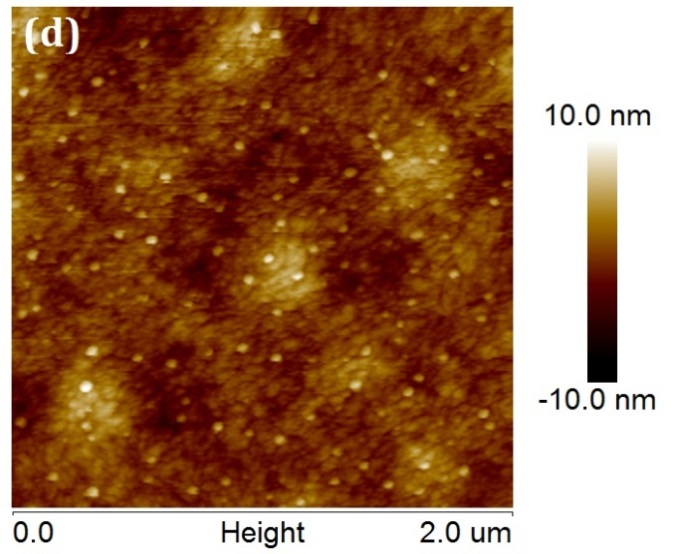


**Fig. S5.** Atomic force microscope images (2 μm × 2 μm) of P3HT films (a) undoped and (b) doped with a 9 mM FeCl_3_/nitromethane solution and PDPP3T films (c) undoped and (d) doped with a 6 mM FeCl_3_/nitromethane solution.

**Fig. S6.** Potential difference ΔV as a function of ΔT between the two electrodes deposited on the PDPP3T film doped with a 6 mM FeCl_3_/nitromethane solution.

**Fig. S7.** Potential difference ΔV as a function of ΔT between the two electrodes deposited on the constantan.

**Fig. S8.** The Seebeck coefficient as a function of electrical conductivity
